# Bighorn sheep gut microbiomes associate with genetic and spatial structure across a metapopulation

**DOI:** 10.1038/s41598-020-63401-0

**Published:** 2020-04-20

**Authors:** Claire E. Couch, Holly K. Arnold, Rachel S. Crowhurst, Anna E. Jolles, Thomas J. Sharpton, Marci F. Witczak, Clinton W. Epps, Brianna R. Beechler

**Affiliations:** 1 Department of Integrative Biology, Oregon State University, 3029 Cordley Hall, Corvallis, OR 97331 USA; 2 Carlson College of Veterinary Medicine, Oregon State University, Magruder Hall, Corvallis, OR 97331 USA; 3 Department of Fisheries and Wildlife, Oregon State University, 104 Nash Hall, Corvallis, OR 97331 USA; 4 Department of Statistics, Oregon State University, 239 Weniger Hall, Corvallis, OR 97331 USA; 5 Department of Microbiology, Oregon State University, 226 Nash Hall, Corvallis, OR 97331 USA

**Keywords:** Community ecology, Microbial ecology

## Abstract

Studies in laboratory animals demonstrate important relationships between environment, host traits, and microbiome composition. However, host-microbiome relationships in natural systems are understudied. Here, we investigate metapopulation-scale microbiome variation in a wild mammalian host, the desert bighorn sheep (*Ovis canadensis nelsoni*). We sought to identify over-represented microbial clades and understand how landscape variables and host traits influence microbiome composition across the host metapopulation. To address these questions, we performed 16S sequencing on fecal DNA samples from thirty-nine bighorn sheep across seven loosely connected populations in the Mojave Desert and assessed relationships between microbiome composition, environmental variation, geographic distribution, and microsatellite-derived host population structure and heterozygosity. We first used a phylogenetically-informed algorithm to identify bacterial clades conserved across the metapopulation. Members of genus *Ruminococcaceae*, genus *Lachnospiraceae*, and family *Christensenellaceae R7 group* were among the clades over-represented across the metapopulation, consistent with their known roles as rumen symbionts in domestic livestock. Additionally, compositional variation among hosts correlated with individual-level geographic and genetic structure, and with population-level differences in genetic heterozygosity. This study identifies microbiome community variation across a mammalian metapopulation, potentially associated with genetic and geographic population structure. Our results imply that microbiome composition may diverge in accordance with landscape-scale environmental and host population characteristics.

## Introduction

Mammals depend on microbial symbionts for extracting and synthesizing nutrients^1^ and training the immune system for pathogen defense^2^. Studies in humans and laboratory animals have demonstrated that the composition of host-associated gut microbiomes is largely determined by a complex interaction of environment, nutrition, and genetics^3–8^, suggesting that the microbiome could mediate relationships between environmental variation and health in natural populations. Laboratory studies are well-suited to experimental manipulation of the microbiome while controlling for the vast majority of genetic and environmental variation that exists in natural populations of mammals, often by using isogenic lines of rodents. It is often challenging, however, to apply results from laboratory studies toward understanding the natural patterns of microbiome composition and structure in genetically diverse host populations exposed to spatial and temporal variability. Additionally, laboratory animals do not generally harbor microbiomes found in their wild counterparts, making it difficult to study the ecology and evolution of many host-microbe relationships^9^. Human studies can span a somewhat wider range of genetic and environmental variation, but these studies are often limited in depth due to the difficulty of collecting important covariates from healthy individuals. In order to understand broad patterns and processes underlying variation in microbiome structure, we need to move beyond controlled laboratory systems and expand to studying microbiomes in natural mammalian populations.

Host-associated microbiomes can be shaped by processes at a range of spatial and temporal scales, influenced by factors such as diet, elevation, climate, and microbial dispersal^10–14^. Wild populations often exhibit strong spatial structuring; some naturally-fragmented populations can be described as metapopulations in which populations experience local extinction and recolonization via dispersal^15,16^. Understanding metapopulation-level microbiome variation in wild hosts has the potential to clarify the ecological and evolutionary processes driving microbiome variation across host scales^17,18^. For example, microbial taxa that are ubiquitous across a metapopulation (i.e. “conserved” microbiota) may be more likely to share an ecological or evolutionary relationship with the host species, or could reflect microbial distribution or dispersal ability^8^. Additionally, metapopulation structure provides multiple population replicates across which to study spatial, demographic, and environmental processes driving microbiome variation between conspecifics^19^.

Environmental variation, host traits, and microbe dispersal ability have been identified as potential drivers of microbiome variation. Host diet is a primary environmental driver of microbiome variation in controlled laboratory studies^13,14^ and humans^3^, and several studies in wild mammals have also demonstrated microbiome variation across spatial and seasonal ranges of nutritional variation. Other environmental factors that may interact with diet to influence the microbiome include altitude^20,21^ and climate^22^. Host factors, such as genetic variation, have been shown to play a role in filtering particular microbes from the environment, thereby influencing the composition and relative abundances of resident microbial communities^23,24^. In addition to genetically-determined host factors, vertical transmission from mother to offspring could also maintain microbiome homogeneity between related hosts^25^. Microbial dispersal between hosts is also thought to drive variation in microbiome diversity and composition. Previous studies have linked microbiome similarity with degree of social interaction within primate populations^26–28^. Some microbes may be environmentally derived rather than directly transmitted between hosts^29^, and in these cases spatial variation in the pool of environmental microbes available to hosts could mediate microbiome stratification between geographically separate host populations. Yet, how microbiome variation and transmission scale across host metapopulations is unknown. Comparing microbiomes across host metapopulations could reveal patterns of variation that clarify mechanisms of selection and dispersal.

Here, we examine microbiome variation across a metapopulation of wild mammals, allowing us to address microbiome variation at a scale previously unexplored. We used gut microbiome data from a naturally fragmented metapopulation of desert bighorn sheep (*Ovis canadensis nelsoni*) in the Mojave Desert of Southern California, which was composed of seven demographically independent and geographically separate populations linked by infrequent dispersal events. This study system enabled us to measure the effects of environmental heterogeneity, geographic proximity, and host genetic diversity and structure on microbiome variation at the metapopulation scale. We framed this study on five central hypotheses & predictions:

*(1) A subset of microbial lineages is conserved across the metapopulation*. Microbes are necessary for nutrient extraction in ruminants^30^, so we hypothesized that host selection would result in the success and ubiquity of certain symbiotic microbiota.

*(2) Environmental differences among habitat patches alter gut microbiome composition*. Previous studies demonstrate shifts in the gut microbiome related to short-term (i.e. seasonal) environmental resource fluctuation^11,31^. We hypothesized that the relationship between microbiome communities and environmental heterogeneity would also manifest on a longer time scale. We compared microbiome community differences with decade-scale summary metrics of three patch-level environmental variables: forage production during the growing season, long-term rainfall levels, and elevation. Decade-scale heterogeneity in vegetation greenness has been shown to correlate with fecal nitrogen levels among Mojave desert bighorn sheep populations^32^, and has been linked to survival in Sierra Nevada populations. Elevation is highly correlated with temperature and forage quality in this desert ecosystem^33,34^, and previous research has suggested that elevation gradients can mediate the relationship between host nutrition and variation in the microbiomes of other mammalian species^20^.

*(3) Population genetic diversity associates with gut microbiome composition*. In our study system, genetic diversity correlates with both elevation and connectivity^34,35^, so we hypothesized that populations with high genetic diversity would differ in terms of presence and abundance of microbial clades relative to low genetic diversity populations.

*(4) Genetically related individuals share similar microbiomes*. Genetic divergence has been previously shown to correlate with microbiome structure among fragmented host populations^24^. We predicted that closely-related individuals would harbor similar gut microbial communities due to host selective processes and vertical transmission.

*(5) Geographic proximity between hosts predicts microbiome similarity*. We hypothesized that spatial proximity of hosts would mediate exposure to similar microbial sources and allow indirect transfer of microbes between hosts^11,12,27^, resulting in microbiome convergence between spatially proximate animals.

## Methods

### Sample collection and DNA extraction

**Figure 1:**
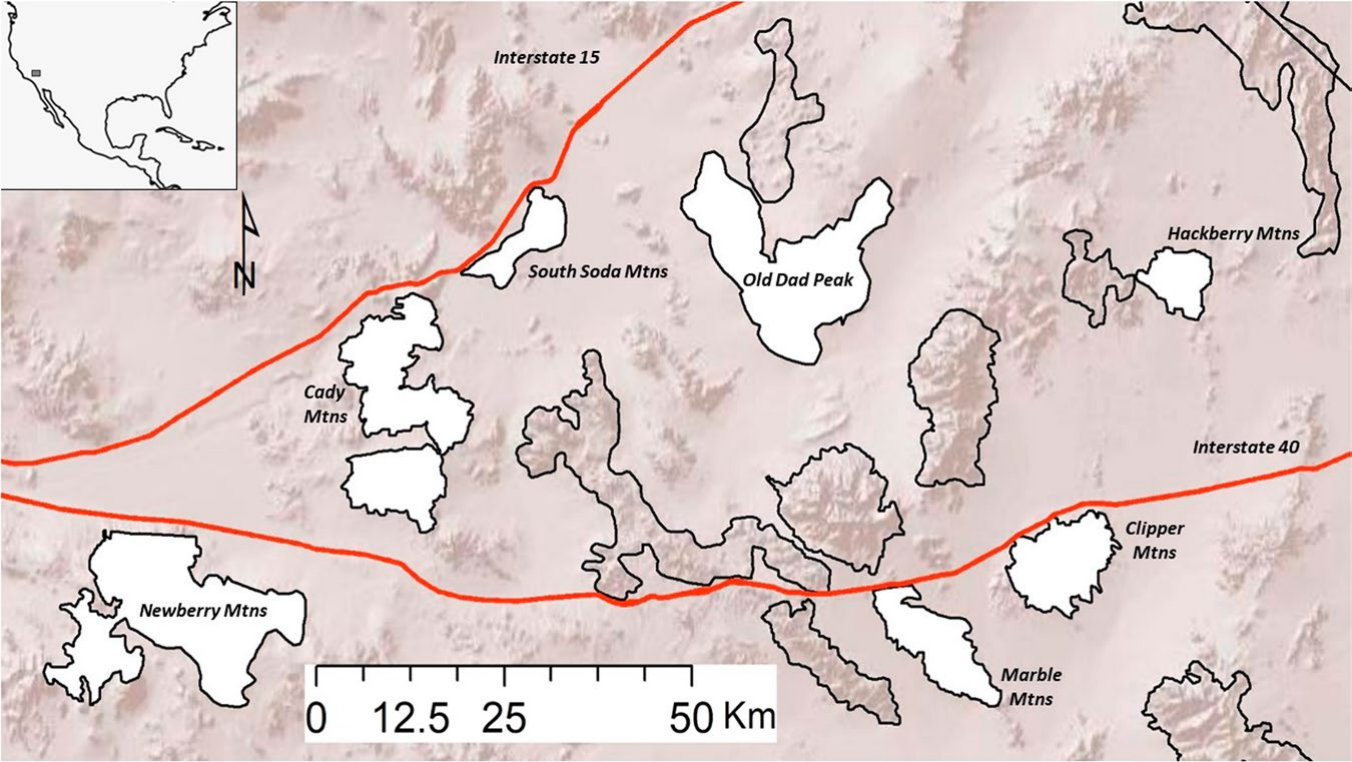
Map of the focal desert bighorn sheep metapopulation in the Mojave Desert of southeastern California. Populations included in this study are colored white and labeled. Other populations are indicated with black outline and shaded backgrounds. The red lines indicate major highways that limit movement between populations. The inset indicates approximate location of the study area within North America.

Fecal samples were collected from seven bighorn sheep populations (11–48 samples per population for host genetics, 3–8 samples per population for microbiome analysis; Table 1) in the southern and central Mojave Desert (Fig. 1). Samples were collected during 2012–2015 by visiting water sources in the summer months (May-August) when bighorn are dependent on water. Fecal samples were assumed to be from lambs and were therefore excluded if they were less than 5 mm in diameter. Sex bias in our sampling was minimal, because male and female bighorn sheep were equally dependent on water sources during the sampling periods. Samples ranged from one day to two weeks old, based on visual assessment of pellets. The extremely dry summer conditions of the Mojave Desert made pre-collection sample contamination and bacterial overgrowth unlikely. Moreover, all samples were dried and stored at room temperature until processing^36^, thus reducing the potential effects of different sample ages. DNA was extracted from samples as described previously^37^. Briefly, DNA was extracted from 30 mg of pellet scrapings using a modified version of the AquaGenomic Stool and Soil protocol (Multitarget Pharmaceuticals, LLC, Colorado Springs, CO) that included 15-minute bead-beating step for cell lysis and the addition of 12 mAU proteinase K (Qiagen Inc., Valencia, CA) to degrade contaminating proteins and nucleases. We added 150 microliters of AquaPrecipi solution (MultiTarget Pharmaceuticals) to cell lysate to remove PCR inhibitors present in fecal samples, and rehydrated DNA pellets overnight in 115 microliters of 1x TE buffer to increase DNA recovery^37^. Duplicate samples from the same individual were excluded from downstream analysis based on genotyping results^37^.

**Table 1:** Summary of environmental and host variables across populations of bighorn sheep in the Mojave Desert, California.

Population	Clipper Mtns	Hackberry Mtns	Cady Mtns	Marble Mtns	Newberry Mtns	Old Dad Peak	South Soda Mtns
Abbreviation	CL	HA	KD	MA	NE	OD	SS
Microbiome sample size	4	6	3	7	8	4	7
Average heterozygosity	0.64	0.63	0.59	0.68	0.52	0.56	0.63
Elevation (meters)	1399	1886	1401	1170	1925	1504	740
NDVI	45.9	55.2	34.2	40.0	39.7	39.9	35.6
Rainfall (millimeters)	190.5	317.5	254.0	190.5	190.5	190.5	254.0
Mean microbiome richness	668	274	555	246	587	555	664

Population abbreviations listed here are used throughout the paper. Expected heterozygosity across 13 microsatellite loci was used to measure genetic diversity of each population. Median values of integrated normalized difference vegetation index (NDVI) from a long-term vegetation study were used to measure differences in long-term potential for forage production in each patch. Mean microbiome richness was calculated for each population based on the number of unique bacterial sequences remaining in each sample after rarefying to account for differences in sequencing depths.

### Host connectivity and genetic diversity

We used pairwise and point measures to describe connectivity and genetic diversity within the bighorn sheep metapopulation in this study. Populations were defined as locally-distributed, demographically-independent groups of bighorn sheep separated from other groups by areas of unsuitable habitat, e.g. flat desert areas lacking escape terrain and suitable food^33,38,39^. The population ranges were defined by the basal contours of each occupied mountain range. Dispersal between populations is limited^40,41^, so these populations function as demographically independent units^33^. To evaluate pairwise genetic structure among individuals, we calculated individual-pairwise Rousset’s a^42^ estimated from 16 neutral microsatellite loci, controlling for genotyping error and allelic dropout as described by Epps *et al*.^37^. Rousset’s a is based on a lattice model, and considers the probability of identity of a gene within an individual compared to probability of identity at a given genetic distance. This metric was selected because it gives accurate measures of distances between individuals within a population, and while at the same time clearly reflecting the strong differentiation between individuals in different populations. For point measures of population-level genetic diversity (Table 1), we calculated population-level expected heterozygosity from 13 of the 16 microsatellite markers used to calculate Rousset’s a. An average of thirty individuals per population (ranging from 11–48 individuals) were used to calculate expected heterozygosity^35^. Three of the 16 markers used in the Rousset’s a calculations were excluded from the expected heterozygosity estimation, because these markers were suspected to be linked with potentially adaptive alleles^43–45^). Although these three loci were shown to behave neutrally^37,46,47^ we chose to exclude these markers from the expected heterozygosity analysis to avoid any cryptic selective influences on heterozygosity metrics. Heterozygosity estimates using a similar number of microsatellite loci have been shown to correlate with population isolation^35^, elevation^34^, and NDVI^32^ in the Mojave Desert bighorn sheep metapopulation.

### Environmental variables

We used decade-scale, patch-level measures of environmental variables previously shown to correlate with bighorn sheep population persistence and diet (Table 1). Elevation is positively correlated with population persistence^33^ and genetic heterozygosity^34^. We interpret this correlation as resulting from the higher water availability and vegetation cover observed at high elevations, which in turn is linked to greater population persistence^33^, and presumably also reflects fewer population bottlenecks during times of drought^34^. Precipitation is likewise correlated with population persistence^33^, as forage growth in this system is strongly precipitation-driven. To define patch-level potential for forage production, we used normalized difference vegetation index data collected from 2000–2011 and integrated over the growing season^32^: briefly, 8-day composite, 250-m resolution NDVI data from the Moderate Resolution Imaging Spectroradiometer (MODIS) data for the years 2000 through 2011 (MOD09Q1, Level 3, Collection 5, tile h08v05) were obtained for all pixels with center points within the boundary of each patch. The median pixel NDVI value was calculated for each patch at every 8-day time point throughout this time period, and the area under the median NDVI curve for each growing season (October 1–June 30) was calculated. This measure of growing season integrated NDVI was shown by Creech *et al*.^32^ to explain the majority of season-level variation in median fecal nitrogen (a proxy for nutrition) in the Marble Mountains and Old Dad Peak populations from within this study system. In this study, we used median integrated NDVI levels from across the 11-year Creech *et al*. study^32^ as a proxy for forage production potential within each patch. In addition to representing forage potential, these values have been demonstrated to correlate with genetic diversity in this system, reflecting multi-generational differences in population size and stability^32^. Geographic distances between individuals were calculated from GPS coordinates of each sample collection site. Elevation for each patch was defined as the highest point occurring in that range, as this indicates the potential for each range to trap precipitation and provide thermal refuges^33,34^.

### PCR and sequencing

We amplified a 450 bp region of the V3/V4 region of the bacterial 16 S gene. Extracted DNA was subject to a first round 16S PCR amplification using the following primers: 16S Forward Primer 5′-TCGTCGGCAGCGTCAGATGTGTATAAGAGA CAGCCTACGGGNGGCWGCAG-3′ and 16 S Reverse Primer 5′-GTCTCGTGGGCTCGGA GATGTGTATAAGAGACAGGACTACHVGGGTATCTAATCC-3′. PCR reactions were amplified with GoTaq Hot Start Polymerase (Promega, Madison, WI) following manufactures suggested use. PCR cycling conditions were as follows: an initial melt of 94 °C for 3 minutes followed by 35 cycles of amplification with a 94 °C for 30 seconds, 55 °C annealing step for 1 minute, and a 68 °C extension step for 1.5 minutes. A final, 5-minute extension step was included following the last cycle. Amplicons were cleaned, indexed, and normalized by the Oregon State University Center for Genome Research and Biocomputing prior to sequencing on the Illumina Miseq v2 platform, resulting in 250 bp paired-end reads.

### Sequence processing

DADA2 (version 1.12.1) was used to identify amplicon sequence variants (ASVs), trim adapter sequences, and remove chimeras^48^. Raw sequence data were processed through the dada2 pipeline using the following trimming parameters: trimLeft = c(17, 21), truncLen = c(250, 250), maxN = 0, maxEE = 2, truncQ = 2. Default parameters were used for estimating error parameters using learnErrors(), and chimeras were removed using removeBimeraDenova (method = “consensus”). Full-length 16S ribosomal RNA sequences were downloaded from the All Species Living Tree Project (SILVA)^49^ and aligned to ASVs obtained above using mothur version 1.39.3. ASVs that did not align well were discarded from further analysis. The Silva database contains a set of highly curated quality rRNA sequences that were used to guide phylogenetic reconstruction of 16S reads. A generalized time-reversible phylogenetic model was constructed from the combined reference and ASV sequences using FastTree version 2.1.10. The phylogenetic tree was midpoint rooted, and reference sequences pruned from the tree. Prior to statistical analyses, samples were rarified to the 11971 reads per sample, deemed appropriate via collector’s curves (Fig. S1).

### Statistical analysis

#### Identification of conserved clades

Many wildlife microbiome studies have sought to identify conserved microbial taxa based on prevalence or abundance thresholds, but this approach risks spuriously misclassifying clades that are widespread simply due to their ancestral position in the bacterial lineage, or are highly derived and do not meet arbitrary prevalence or abundance cutoffs. We applied a recently-developed bioinformatic algorithm^8^ to identify clade-based taxonomic units. In brief, this algorithm identifies conserved monophyletic clades of taxa among groups (here, bighorn sheep populations) which displayed higher prevalence across the group of interest than expected by chance, based on that clade’s position in a phylogenetic tree. The computational procedure traverses a phylogeny assembled from 16S rRNA gene sequences generated from multiple communities. It then quantifies each clade’s prevalence across a defined subset of the communities, where the clade’s prevalence is based on the occurrence of the subtending lineages in the subset of communities. A permutation test quantifies whether the observed prevalence of the clade is likely due to chance. The algorithm then assigns taxonomic labels to each node in the phylogeny by determining the most specific taxonomic assignment that is shared between all subtending lineages of that clade. We used this algorithm with n = 1,000 permutations to identify monophyletic clades of gut bacteria that were more prevalent than expected by chance within individual host populations and across all individuals in the entire metapopulation (referred to hereafter as “conserved”) based on false discovery-rate adjusted p-values (FDR). Conserved microbiota within and between populations were visualized using ggtree version 1.12.7^50^ and R version 3.5.0.

#### Alpha diversity and compositional analyses

ASV richness (number of unique bacterial sequences) was calculated from rarefied count data. A Kruskal-Wallace test was used to compare ASV richness between populations. Weighted unifrac and Jaccard distances were calculated between all samples to be used for downstream multivariate analyses^51^. The adonis function in the R vegan package (version 2.5.5) was used to conduct pairwise permutational multivariate analysis of variance (PERMANOVA) testing for significant compositional differences between populations, and the betadisper function was used to test differences in multivariate dispersion between populations^52^. Nonmetric multidimensional scaling (NMDS) of weighted unifrac distances was performed with the vegan function metaMDS() and used to visualize compositional differences between individuals and populations.

#### Geographic, genetic, & environmental distance analyses

To assess how microbiome similarity correlated with geographic/genetic distances and environmental variables, we used the lmer function in the lme4 package to run linear mixed-effects models comparing pairwise weighted unifrac and Jaccard (presence/absence) distances with (1) pairwise individual-level geographic distances and Rousset’s a, and (2) pairwise differences in population-level traits (heterozygosity, elevation, NDVI, and rainfall) as fixed effects, and population memberships as random effects^53^. To test our hypotheses, we compared increasingly reduced versions of models 1(a), 1(b), 2(a), and 2(b) for both weighted unifrac and Jaccard distances using sequential chi-squared tests and confirmed our model selection results by comparing AIC and BIC values (See Supplementary Table S4 for model selection details).

1a $$\begin{array}{c}<mml:maligngroup xmlns:xlink="http://www.w3.org/1999/xlink"/>{\rm{Weighted}}\,{\rm{Unifrac}}\,{{\rm{Distance}}}_{{\rm{ij}}} \sim {\rm{Rousset}}\mbox{'}{\rm{s}}\,{{\rm{a}}}_{{\rm{ij}}}\,\ast \,{\rm{Geographic}}\,{{\rm{Distance}}}_{{\rm{ij}}}+(1|{{\rm{Population}}}_{{\rm{i}}})\\ <mml:maligngroup xmlns:xlink="http://www.w3.org/1999/xlink"/>\,+(1|{{\rm{Population}}}_{{\rm{j}}})\end{array}$$

1b $$\begin{array}{c}<mml:maligngroup xmlns:xlink="http://www.w3.org/1999/xlink"/>{\rm{Jaccard}}\,{{\rm{Distance}}}_{{\rm{ij}}} \sim {\rm{Rousset}}\mbox{'}{\rm{s}}\,{{\rm{a}}}_{{\rm{ij}}}\,\ast \,{\rm{Geographic}}\,{{\rm{Distance}}}_{{\rm{ij}}}+(1|{{\rm{Population}}}_{{\rm{i}}})\\ <mml:maligngroup xmlns:xlink="http://www.w3.org/1999/xlink"/>\,+(1|{{\rm{Population}}}_{{\rm{j}}})\end{array}$$

2a $$\begin{array}{c}<mml:maligngroup xmlns:xlink="http://www.w3.org/1999/xlink"/>{\rm{Weighted}}\,{\rm{Unifrac}}\,{{\rm{Distance}}}_{{\rm{ij}}} \sim {{\rm{NDVI}}}_{{\rm{i}}-{\rm{j}}}+{{\rm{Heterozygosity}}}_{{\rm{i}}-{\rm{j}}}+{{\rm{Elevation}}}_{{\rm{i}}-{\rm{j}}}\\ <mml:maligngroup xmlns:xlink="http://www.w3.org/1999/xlink"/>+{{\rm{Rainfall}}}_{{\rm{i}}-{\rm{j}}}\,+(1|{{\rm{Population}}}_{{\rm{i}}})+(1|{{\rm{Population}}}_{{\rm{j}}})\end{array}$$

2b $$\begin{array}{c}<mml:maligngroup xmlns:xlink="http://www.w3.org/1999/xlink"/>{\rm{Jaccard}}\,{{\rm{Distance}}}_{{\rm{ij}}} \sim {{\rm{NDVI}}}_{{\rm{i}}-{\rm{j}}}+{{\rm{Heterozygosity}}}_{{\rm{i}}-{\rm{j}}}+{{\rm{Elevation}}}_{{\rm{i}}-{\rm{j}}}+{{\rm{Rainfall}}}_{{\rm{i}}-{\rm{j}}}\\ <mml:maligngroup xmlns:xlink="http://www.w3.org/1999/xlink"/>\,+(1|{{\rm{Population}}}_{{\rm{i}}})+(1|{{\rm{Population}}}_{{\rm{j}}})\end{array}$$

## Results

### Hypothesis 1: A subset of microbial lineages is conserved across the host metapopulation

**Figure 2:**
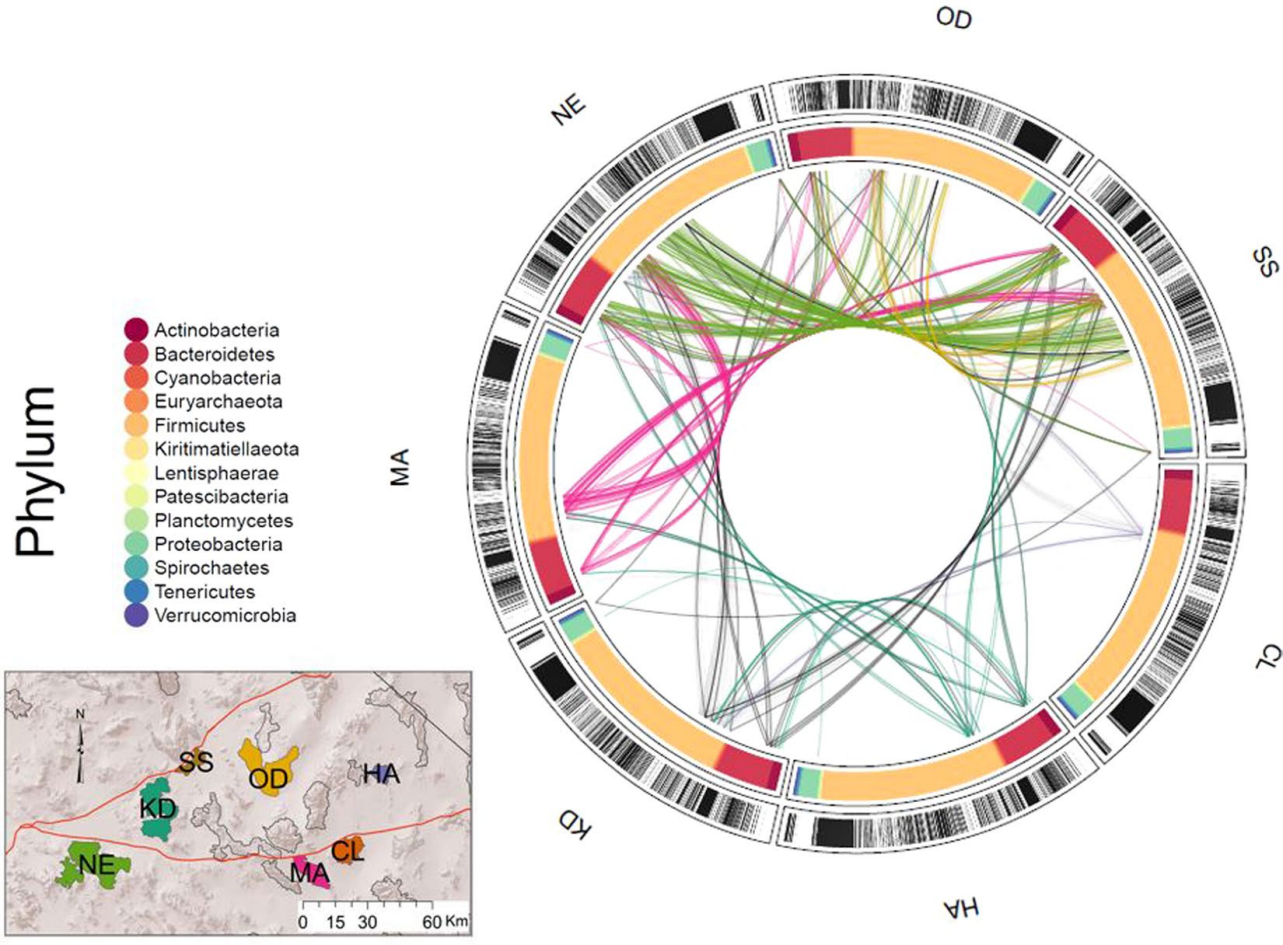
A subset of bacterial clades were conserved within the host metapopulation. Permutation tests were used to identify clades that were overrepresented within the entire metapopulation, and for each separate population (n permutations = 1,000). Each of the seven segments of the circle indicates one of the seven bighorn populations. The inner ring indicates phylum-level classification of each clade that was conserved in the metapopulation and/or in one or more subpopulations. Clades were excluded from the figure if they did not meet these criteria. A black bar in the outer ring indicates that a particular clade was conserved in that population, and a white bar indicates the clade was not conserved in that population. Linear models were used to identify clades that were potentially shared via environmental overlap or population interactions. Colored arced lines connecting populations indicate clades that are shared between two populations and are associated with geographic proximity (therefore potentially shared via environmental overlap). Colors are mapped to population colors in Fig. 1. Black lines indicate clades that are associated with genetic distance and therefore potentially shared via host-host interactions.

We found that 894 out of 13474 total clades (6.6%) were conserved across the host metapopulation, meaning that they were more frequently observed among hosts than would be expected based on their position in the bacterial phylogeny (FDR < 0.01). These conserved clades corresponded to 80 unique bacterial taxa (identified via alignment with the SILVA database). Conserved clades tended to be “nested” meaning that a conserved clade was likely to also have a descendant sub-clade in the phylogeny that was also conserved. We considered that this pattern of nested conserved clades could result from propagation of the signal of clade conservation between such directly related clades, which would have the effect of artificially inflating the number of apparently conserved clades. We corrected for this possibility by only considering the most ancestral conserved clade among a set of directly related clades that were consistently identified as being conserved. This correction for nestedness resulted in a conservative set of 270 clades corresponding to 67 unique taxa (Fig. 2; Supplementary Table S1). The taxa containing the highest number of conserved clades were *Ruminococcaceae* (family; 99 conserved clades), *Ruminococcaceae UCG 005* (genus; 93 conserved clades), *Ruminococcaceae UCG 010* (genus, 88 conserved clades), *Christensenellaceae R7 group* (genus, 76 conserved clades), and *Lachnospiraceae* (family, 58 conserved clades).

Although some bacterial clades were conserved across the entire metapopulation, analyzing each population separately demonstrated that bighorn sheep populations harbor distinct conserved clades of gut microbiota. Permutation tests applied individually to each of the seven host populations revealed differing numbers and identities of conserved clades within each population (Supplementary Figs. S2 and S3), in addition to differences in the number of bacterial clades shared between population pairs. In total, there were 1194 unique bacterial clades that were conserved within one or more host populations. The number of clades conserved within each population varied from 81 (CL) to 499 (SS) before correction for nestedness, and 55 (MA) to 238 (SS) after correction for nestedness (Supplementary Fig. S2). Among unnested conserved clades, 31% were conserved in more than one population, suggesting that they have an important relationship with the host or are widespread in the environment. No clades were found to be conserved within all of the populations, but a single clade was conserved in 6 of the 7 populations. This clade was taxonomically labeled as family *Lachnospiraceae*, and contained only 3 descendent ASVs. Eleven clades were conserved in five or more populations, including members of family *Lachnospiraceae*, family *Ruminococcaceae*, genus *Bacteroides*, order *Lactobacillales*, and genus *Coprococcus* (Table S2).

### Hypotheses 2 & 3: Host genetic heterozygosity, but not environmental variation, exhibits possible correlations with gut microbiome composition

**Figure 3:**
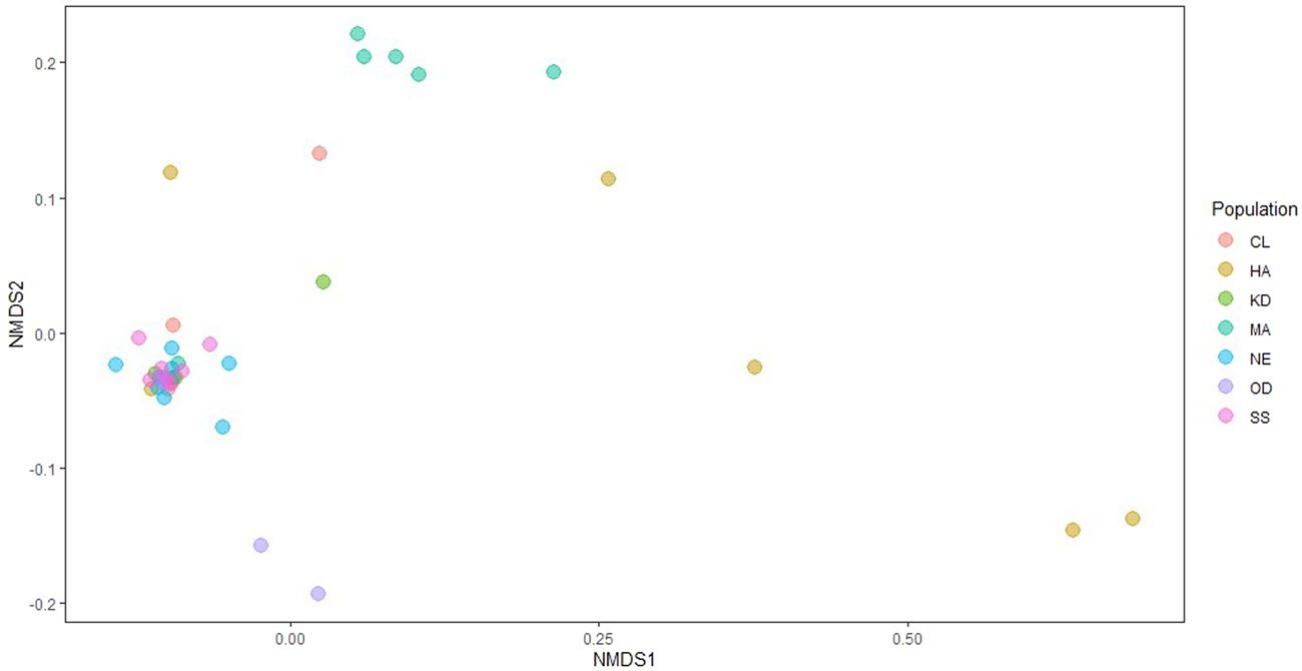
Bighorn gut microbiome communities differ among populations. Nonmetric multidimensional scaling was applied to weighted unifrac distances between microbiome communities within each host. Points indicate the relative locations of individual hosts within microbiome space, colored by population.

Analysis of microbial alpha diversity failed to demonstrate significant metapopulation-level structure in terms of microbial richness (Table 1, Supplementary Fig. S4). However, beta diversity analysis demonstrated inter-population differences in composition between some population pairs (Supplementary Table S3). Pairwise PERMANOVA tests comparing weighted unifrac distances revealed significant differences in microbiome ASV composition (FDR < 0.05) between five of the 21 pairs of populations, and this result was confirmed by visualizing inter-sample similarities in an NMDS plot (Fig. 3, Supplementary Table S3). Linear mixed-effects models demonstrated significant, positive relationships between similarity in microbiome composition and similarity in genetic heterozygosity (Table S4). Model selection for the weighted unifrac model (2a) resulted in a final model containing only heterozygosity as a fixed effect (Eq. 3a), and selection on the Jaccard model resulted in a model containing heterozygosity and NDVI (Eq. 3b). However, the only term that was significant at the p < 0.01 level was heterozygosity in Eq. 3(b), which demonstrated an association between Jaccard distance and difference in heterozygosity after controlling for NDVI (estimate = 0.013, p = 1.96e-06).

3a $${\rm{W}}{\rm{e}}{\rm{i}}{\rm{g}}{\rm{h}}{\rm{t}}{\rm{e}}{\rm{d}}\,{\rm{U}}{\rm{n}}{\rm{i}}{\rm{f}}{\rm{r}}{\rm{a}}{\rm{c}}\,{{\rm{D}}{\rm{i}}{\rm{s}}{\rm{t}}{\rm{a}}{\rm{n}}{\rm{c}}{\rm{e}}}_{{\rm{i}}{\rm{j}}}\sim {{\rm{H}}{\rm{e}}{\rm{t}}{\rm{e}}{\rm{r}}{\rm{o}}{\rm{z}}{\rm{y}}{\rm{g}}{\rm{o}}{\rm{s}}{\rm{i}}{\rm{t}}{\rm{y}}}_{{\rm{i}}-{\rm{j}}}+(1|{{\rm{P}}{\rm{o}}{\rm{p}}{\rm{u}}{\rm{l}}{\rm{a}}{\rm{t}}{\rm{i}}{\rm{o}}{\rm{n}}}_{{\rm{i}}})+(1|{{\rm{P}}{\rm{o}}{\rm{p}}{\rm{u}}{\rm{l}}{\rm{a}}{\rm{t}}{\rm{i}}{\rm{o}}{\rm{n}}}_{{\rm{j}}})$$

3b $${\rm{J}}{\rm{a}}{\rm{c}}{\rm{c}}{\rm{a}}{\rm{r}}{\rm{d}}\,{{\rm{D}}{\rm{i}}{\rm{s}}{\rm{t}}{\rm{a}}{\rm{n}}{\rm{c}}{\rm{e}}}_{{\rm{i}}{\rm{j}}}\sim {{\rm{H}}{\rm{e}}{\rm{t}}{\rm{e}}{\rm{r}}{\rm{o}}{\rm{z}}{\rm{y}}{\rm{g}}{\rm{o}}{\rm{s}}{\rm{i}}{\rm{t}}{\rm{y}}}_{{\rm{i}}-{\rm{j}}}+{\rm{N}}{\rm{D}}{\rm{V}}{\rm{I}}+(1|{{\rm{P}}{\rm{o}}{\rm{p}}{\rm{u}}{\rm{l}}{\rm{a}}{\rm{t}}{\rm{i}}{\rm{o}}{\rm{n}}}_{{\rm{i}}})+(1|{{\rm{P}}{\rm{o}}{\rm{p}}{\rm{u}}{\rm{l}}{\rm{a}}{\rm{t}}{\rm{i}}{\rm{o}}{\rm{n}}}_{{\rm{j}}})$$

### Hypotheses 4 & 5: Geographic proximity and genetic connectivity associate with different metrics of microbiome similarity between populations

Linear mixed-effects models showed that geographic proximity and genetic relatedness differed in their effects, depending which microbiome distance metric was used. Model selection on models 1(a) and 1(b) yielded different results based on the microbiome distance used (Eq. 4a,b).

4a $${\rm{Weighted}}\,{\rm{Unifrac}}\,{{\rm{Distance}}}_{{\rm{ij}}} \sim {\rm{Rousset}}\mbox{'}{\rm{s}}\,{{\rm{a}}}_{{\rm{ij}}}+(1|{{\rm{Population}}}_{{\rm{i}}})+(1|{{\rm{Population}}}_{{\rm{j}}})$$

4b $${\rm{Jaccard}}\,{{\rm{Distance}}}_{{\rm{ij}}} \sim {\rm{Geographic}}\,{{\rm{Distance}}}_{{\rm{ij}}}+(1|{{\rm{Population}}}_{{\rm{i}}})+(1|{{\rm{Population}}}_{{\rm{j}}})$$

Jaccard distance was significantly associated with geographic proximity (estimate = 0.0185, p = 2.69e-7) but not Rousset’s a, whereas weighted unifrac distance was significantly associated with Rousset’s distance (estimate = 0.0196, p = 0.00316). The total proportion of bacterial clades that associated positively with geographic proximity was 49.2%, including nested clades (Fig. 2). Of these clades, 178 (1.3%) were significantly associated with geographic proximity (FDR < 0.05).

## Discussion

Our findings reveal microbiome composition structure at the metapopulation scale in a natural wildlife system. We identified bacterial lineages conserved across a bighorn sheep metapopulation, despite differences between populations in terms of microbiome composition. At the population level, differences in microbiome communities correlated with genetic heterozygosity, suggesting that intrinsic host factors play a role in microbial composition even at a metapopulation scale. At the individual level, geographic proximity was a significant predictor of the presence or absence of microbial ASVs, and genetic relatedness was not significant. However, the effect of genetic relatedness dominated the effect of geographic proximity when using weighted unifrac distances, which incorporate phylogenetic relatedness and relative abundance of ASVs. These findings suggest that home range overlap mediates microbial exposure, whereas host genetics mediates selection of different microbial lineages. However, further research is needed to fully disentangle the effects of inheritance and social grouping on shaping the bighorn sheep microbiome.

We identified microbial clades that were overrepresented across the metapopulation, supporting the hypothesis that host selection results in the success and ubiquity of certain symbiotic microbiota. The bacterial taxa containing the largest numbers of conserved clades belonged to family *Ruminococcaceae*, family *Lachnospiraceae*, and genus *Christensenellaceae R7 group*, all of which are members of order *Clostridiales* and phylum *Firmicutes*. Families *Ruminococcaceae* and *Lachnospiraceae* are dominant fecal bacterial families in domestic sheep^54^, and all three taxa are known to be important rumen symbionts that associate positively with consumption of high-forage diets^55,56^. Members of family *Ruminococcaceae* are known to play an important role in initiating the breakdown of plant fiber in the rumen. Members of *Ruminococcaceae* and *Lachnospiraceae* may be responsible for biohydrogenation in the rumen, converting dietary poly-unsaturated fatty acids to saturated fatty acids^57^. Genus *Christensenellaceae R7 group* belongs to family *Christensenellaceae*, a highly heritable taxon in the human gut microbiome that may have adaptive significance for host metabolism^58^. Although the rumen microbiome is structurally distinct from the fecal microbiome in domestic sheep^59^, many of the same taxonomic groups of microbes are present at these two sites, and differences in feed efficiency are reflected in community changes to both the ruminal and fecal microbiomes. Notably, members of families *Ruminococcaceae* and *Christensenellaceae* are relatively enriched in the feces of domestic lambs with high feed efficiency versus those with low feed efficiency, and ruminal *Lachnospiraceae* are negatively associated with feed efficiency. Some bacterial lineages may be conserved because they are ubiquitous in the environment or are highly adept at dispersal; however, based on their inferred relationship with ruminant nutrition^55–57,59^ and (in the case of *Christensenellaceae*) host heritability^58^, we propose that family *Ruminococcaceae*, family *Lachnospiraceae*, and genus *Christensenellaceae R7 group* are conserved across bighorn sheep populations due to an adaptive relationship with their host.

The positive association observed between geographic distance and shared presence/absence of microbial ASVs supports the hypothesis that that spatial proximity of hosts mediates exposure to similar microbial sources and allows indirect transfer of microbes between animals^11,12,27^. Moreover, the significant positive relationship between weighted unifrac distance and inter-individual genetic distance implies that genetically similar (and thus closely-related) individuals exert similar selective pressures on their gut microbes, resulting in similar abundances of phylogenetically related microbes among closely-related hosts. Alternatively, closely-related individuals may share similar microbiomes due to vertical transmission from mother to offspring, or because related individuals tend to associate in social groups. Although this observational study included a limited number of populations and cannot fully disentangle the influences of geographic and genetic distance on microbiome variation, the observed association between geographic proximity and microbiome similarity indicate that dispersal limitation could play an important role in microbiome divergence between populations. Studies in humans have demonstrated microbiome variation across large scales between geographically distinct populations^60–62^. Wildlife studies^11,12,27^ demonstrated spatial patterns of gut microbiome composition within wildlife populations, and our study suggests that this pattern holds true at the metapopulation scale. Further research is needed to fully parse the relative roles of genetics, vertical transmission, and spatial/social structure on microbiome assembly.

At the host population level, we found a positive, significant relationship between microbiome divergence and differences in population-level genetic heterozygosity, suggesting alternative patterns of microbial composition in populations with high versus low heterozygosity. However, we found no relationship between microbiome beta diversity and long-term measures of patch-scale environmental variation. This result was somewhat surprising, as a growing body of literature points to nutritional resource availability^11,31,63–65^ and other environmental conditions such as elevation^20,21^ as primary drivers of microbiome community structure among conspecifics. However, the temporal or spatial scales at which we measured environmental variation may not have been fine enough to detect correlations with microbiome variation, or possibly sample sizes for each population were insufficient to detect relationships between patch-level environmental variables and microbiome structure. Our study used decade-long, patch-scale measures of forage production and rainfall, and patch-level elevation maxima, but future studies should include finer-scale environmental data. The relationship we observed between microbiome divergence and difference in population-level genetic heterozygosity also may have been limited by a relatively small number of populations. Previous studies in laboratory and wildlife studies have shown microbiome differences related to MHC genotype diversity^66–68^, but larger scale assessments are necessary to elucidate the role of neutral genetic heterozygosity as a potential driver of metapopulation-scale variation in the microbiome in bighorn sheep.

Our findings demonstrate that microbiome variation aligns with host genetic and spatial structure in a wild mammalian metapopulation. In addition to the broad implications for understanding the ecology of mammalian microbiomes, this is also the first study to describe the bighorn sheep gut microbiome, or to identify potential drivers of gut microbiome composition and diversity in this culturally iconic species. Ultimately, understanding the dynamics and ecology of the bighorn sheep gut microbiome could have implications for population health and conservation^16^, therefore future studies should seek to further disentangle the metapopulation-scale effects of environmental variation, microbial dispersal, and host selection on the microbiome, and evaluate links between the gut microbiome and infectious disease susceptibility. The small number of samples from some populations may have limited the number of conserved clades we were able to detect in this analysis, thus future studies that seek to define conserved microbial clades should include more samples from each population. Additionally, increased depth of sequencing could improve detection of low-abundance conserved clades. Broadly, our findings contribute to understanding the underlying variation of host-associated microbiomes at the metapopulation scale, and specifically inform our understanding of gut microbiome communities of a culturally iconic herbivore species.

## Supplementary information

Supplementary Information.
