# Supplementary material for: Bighorn sheep gut microbiomes associate with genetic and spatial structure across a metapopulation
Source: Sci Rep. 2020 Apr 20;10:6582. doi: 10.1038/s41598-020-63401-0 (PMC7171152; doi:10.1038/s41598-020-63401-0)
Supplement: Supplementary file 1 — Supplementary Information. [file 41598_2020_63401_MOESM1_ESM.docx]

Supplementary Information

Manuscript title: Bighorn sheep gut microbiomes associate with genetic and spatial structure across a metapopulation.

Short title: Metapopulation-scale variation in the bighorn sheep gut microbiome.

Authors:

Claire E. COUCH*^1^ Holly K. ARNOLD^^2^ Rachel S. CROWHURST^3^ Anna E. JOLLES^4^ Thomas J. SHARPTON^5^ Marci F. WITCZAK^2^ Clinton W. EPPS+^3^, Brianna R. BEECHLER+^4^

*indicates corresponding author

^indicates shared first authorship

+indicates shared senior authorship


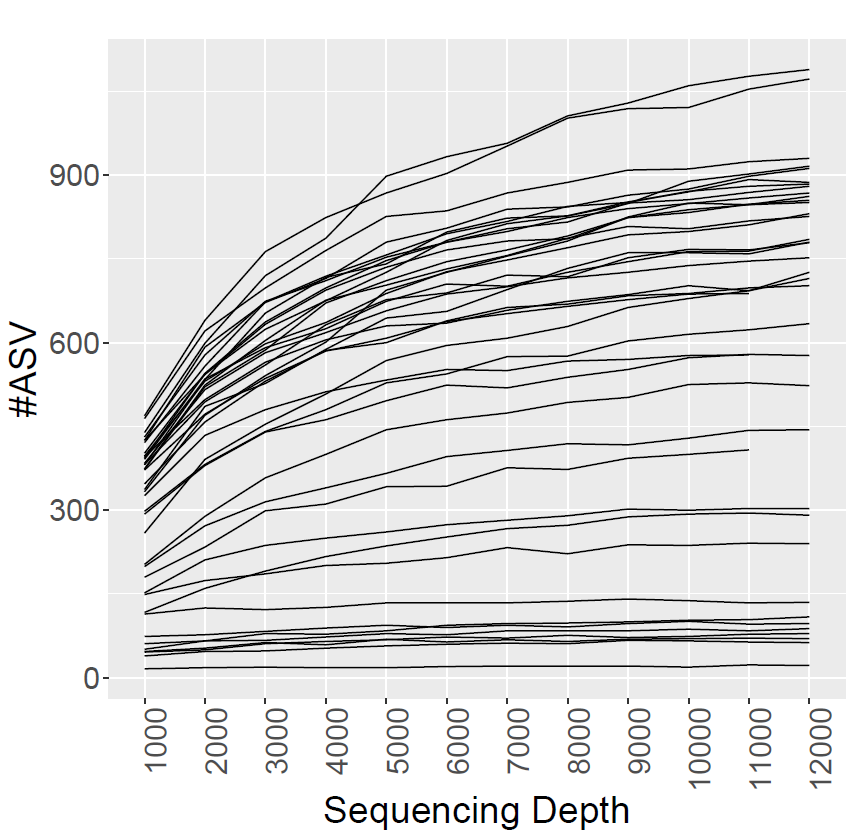


Figure S1: Collector’s curves for each sample were generated based on rarefaction by random subsampling without replacement at 1000-read intervals from 1,000-12,000 reads per sample. Reads were randomly sampled without replacement to determine how many unique sequences were lost at each step. Collector’s curves demonstrate that saturation of ASVs was achieved for all samples near 10,000 reads, and therefore samples were rarified to 11,971 (the minimum sample depth) prior to downstream analysis.

Table S1: Taxonomic labels of un-nested CTUs conserved across all sheep populations. CTUs p-values were obtained by randomly permuting (n = 1000) the tree tip-to-ASV labels, and comparing the observed conservation value to the the shu_ed conservation values. P-values were corrected using a false discovery rate correction (q < 0:01). CTUs were removed if they had an ancestor that was also conserved. Each CTU was annotated with a taxonomic label corresponding to the lowest taxonomic level that was shared by all members within the clade. The lowest taxonomic level is shown on the right. The letter corresponds to what level it is at (K - kingdom; P - phylum; C - class; O - order; F - family; G - genus). The number in the right column corresponds to how many clades were discovered which shared this taxonomic level.


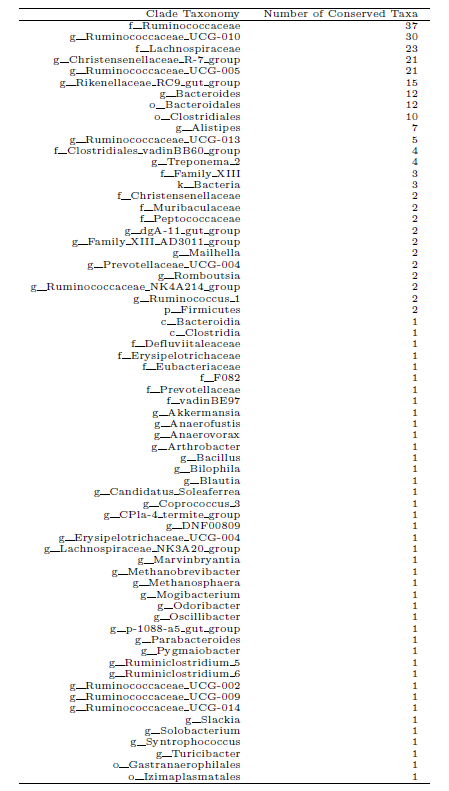


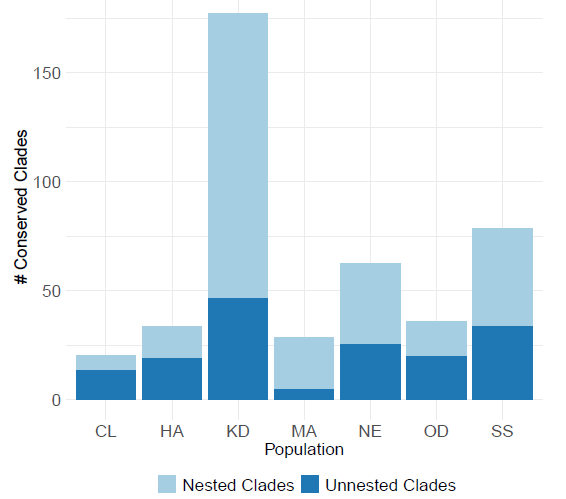


Figure S2: A group permutation test was run for each clade for each population (n = 1000). A false discovery rate correction was applied (q < 0.05). The average number of conserved clades for each individual (y-axis) is shown for each population (x-axis). Before (light blue) and after correction for nesting (dark blue) is shown.


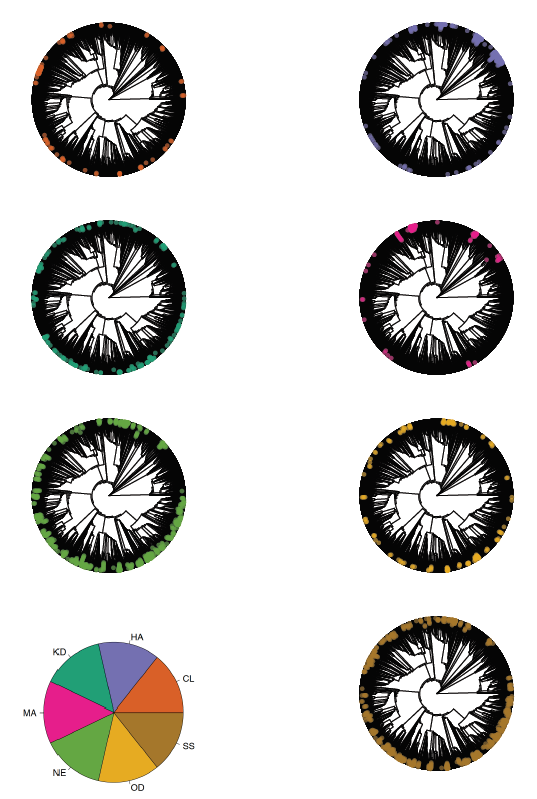


Figure S3: Host populations showed different patterns of conserved microbial clades. A group permutation test was run for ech clade within each population (n = 1,000), and a false discovery rate correction was applied to identify clades that were significantly conserved within each population (q < 0.05). In order to visualize how conserved clades were phylogenetically clustered within populations, these clades were mapped to the entire bacterial phylogenetic tree. Some populations contained taxonomically grouped “hotspots” on the phylogenetic tree.

Table S2: Taxonomic labels of clades conserved in 5 of the populations.


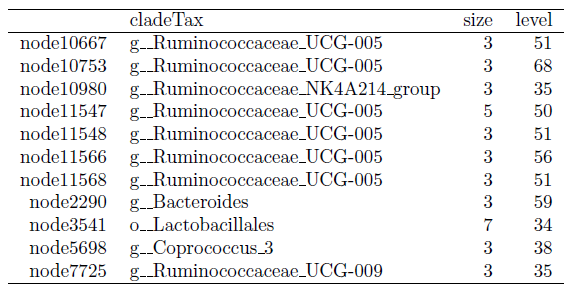


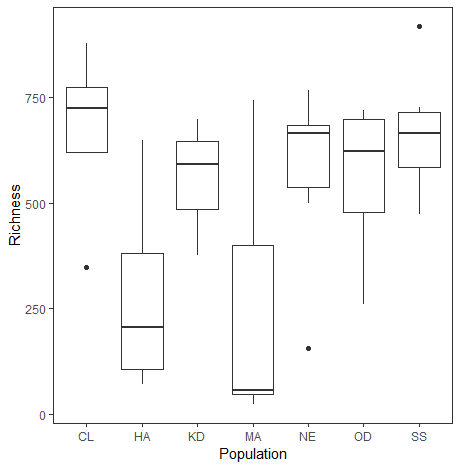


Figure S4: Box and whisker plots display the following summary values for ASV richness: median (horizontal line dissecting box), upper and lower quartiles (top and bottom box edges), highest and lowest data values that fall within 1.5 times the interquartile distance from the box edges (lines extending to “whiskers”), and data outliers outside of this range (circles). Population abbreviations are listed in Table 1.

Table S3: Significant pairwise differences in microbiome community composition were observed between some bighorn sheep populations. Pairwise PERMANOVA was used to compare compositional differences between populations using weighted unifrac distances, and PERMDISP2 was used to compare multivariate dispersions. The upper triangle contains p-values for pairwise homogeneity of multivariate dispersion, and the lower triangle of the table shows p-values for the pairwise community composition comparisons. P-values were corrected for multiple testing using the Benjamini-Hochberg adjustment. Significant (false discovery rate-adjusted q <0.05) results are indicated with an asterisk. See Table 1 for population abbreviations.

| Population | CL | HA | MA | NE | OD | SS | KD |
| --- | --- | --- | --- | --- | --- | --- | --- |
| CL |  | 0.147 | 0.668 | 0.668 | 0.668 | 0.480 | 0.964 |
| HA | 0.138 |  | 0.301 | 0.011* | 0.147 | 0.011* | 0.147 |
| MA | 0.138 | 0.095 |  | 0.379 | 0.889 | 0.273 | 0.668 |
| NE | 0.088 | 0.021* | 0.037* |  | 0.273 | 0.380 | 0.668 |
| OD | 0.202 | 0.138 | 0.095 | 0.132 |  | 0.147 | 0.668 |
| SS | 0.131 | 0.014* | 0.046* | 0.014* | 0.138 |  | 0.380 |
| KD | 0.604 | 0.202 | 0.202 | 0.557 | 0.449 | 0.182 |  |

Table S4: Model selection results, AIC, BIC, and rankings for models 1 & 2. Model 1 evaluated associations with microbiome distance and genetic/geographic distances. Model 2 evaluated associations with differences in environmental and population traits. Chi-squared tests were used to compare progressively more complex (forward selection) or reduced (backward selection) models to identify the models that best fit the data. AIC and BIC scores were also calculated and compared to confirm model selection results.

| Model | Geographic & Genetic Distance Models | | Environmental and Population Traits Models | |
| --- | --- | --- | --- | --- |
|  | Weighted unifrac | Jaccard | Weighted unifrac | Jaccard |
| Full model | *Wunifrac_ij_ ~ Rousset’s a_ij_* Geographic Distance_ij_ + (1\|Population_i_) + (1\|Population_j_)*  *AIC = -511.27*  *BIC = -486.41* | *Jaccard_ij_ ~ Rousset’s a_ij_* Geographic Distance_ij_ + (1\|Population_i_) + (1\|Population_j_)*  *AIC = -1171.2*  *BIC = -1142.2* | *Wunifrac Distance_ij_ ~ NDVI_i-j_ + Heterozygosity_i-j_ + Elevation_i-j_ + Rainfall_i-j_ +  (1\|Population_i_) + (1\|Population_j_)*  *AIC = -506.05*  *BIC = -472.91* | *Jaccard Distance_ij_ ~ NDVI_i-j_ + Heterozygosity_i-j_ + Elevation_i-j_ + Rainfall_i-j_ +  (1\|Population_i_) + (1\|Population_j_)*  *AIC = -1169.4*  *BIC = 1136.3* |
| Null model | *Wunifrac_ij_ ~ (1\|Population_i_) + (1\|Population_j_)*  *AIC = -505.06*  *BIC = -488.49* | *Jaccard_ij_ ~ (1\|Population_i_) + (1\|Population_j_)*  *AIC = -1145.5*  *BIC = -1129.0* | *Wunifrac Distance_ij_ ~ (1\|Population_i_) + (1\|Population_j_)*  *AIC = -505.06*  *BIC = -488.49* | *Jaccard Distance_ij_ ~ (1\|Population_i_) + (1\|Population_j_)*  *AIC = -1145.5*  *BIC = -1129.0* |
| Forward selection result | *Wunifrac_ij_ ~ Rousset’s a_ij_ + (1\|Population_i_) + (1\|Population_j_)*  *AIC = -511.86*  *BIC = -491.15* | *Jaccard_ij_ ~ Geographic Distance_ij_ + (1\|Population_i_) + (1\|Population_j_)*  *AIC = -1170.8*  *BIC = -1150.1* | *Wunifrac Distance_ij_ ~ Heterozygosity_i-j_ + (1\|Population_i_) + (1\|Population_j_)*  *AIC = -509.41*  *BIC = -488.70* | *Jaccard Distance_ij_ ~ NDVI_i-j_ + Heterozygosity_i-j_ + (1\|Population_i_) + (1\|Population_j_)*  *AIC = -1172.4*  *BIC = 1147.5* |
| Backward selection result | *Wunifrac_ij_ ~ Rousset’s a_ij_ + (1\|Population_i_) + (1\|Population_j_)*  *AIC = -511.86*  *BIC = -498.15* | *Jaccard_ij_ ~ Geographic Distance_ij_ + (1\|Population_i_) + (1\|Population_j_)*  *AIC = -1170.8*  *BIC = -1150.1* | *Wunifrac Distance_ij_ ~ Heterozygosity_i-j_ + (1\|Population_i_) + (1\|Population_j_)*  *AIC = -509.41*  *BIC = -488.70* | *Jaccard Distance_ij_ ~ NDVI_i-j_ + Heterozygosity_i-j_ + (1\|Population_i_) + (1\|Population_j_)*  *AIC = -1172.4*  *BIC = 1147.5* |
| Significance of final model fixed effects | Rousset’s a (estimate = 0.0196, p = 0.00316) | Geographic distance (estimate = 0.0184, p = 2.69e-07) | Heterozygosity (estimate = 0.0198, p = 0.0107) | NDVI (estimate = 0.0125, p = 0.0127)  Heterozygosity (estimate = 0.0178, p = 1.92e-06) |
